# A Brain-Machine Interface for Control of Medically-Induced Coma

**DOI:** 10.1371/journal.pcbi.1003284

**Published:** 2013-10-31

**Authors:** Maryam M. Shanechi, Jessica J. Chemali, Max Liberman, Ken Solt, Emery N. Brown

**Affiliations:** 1 School of Electrical and Computer Engineering, Cornell University, Ithaca, New York, United States of America; 2 Department of Electrical Engineering and Computer Science, University of California, Berkeley, California, United States of America; 3 Department of Anesthesia, Critical Care and Pain Medicine, Massachusetts General Hospital, Boston, Massachusetts, United States of America; 4 Institute for Medical Engineering and Science, Massachusetts Institute of Technology, Cambridge, Massachusetts, United States of America; 5 Department of Brain and Cognitive Sciences, Massachusetts Institute of Technology, Cambridge, Massachusetts, United States of America; Indiana University, United States of America

## Abstract

Medically-induced coma is a drug-induced state of profound brain inactivation and unconsciousness used to treat refractory intracranial hypertension and to manage treatment-resistant epilepsy. The state of coma is achieved by continually monitoring the patient's brain activity with an electroencephalogram (EEG) and manually titrating the anesthetic infusion rate to maintain a specified level of burst suppression, an EEG marker of profound brain inactivation in which bursts of electrical activity alternate with periods of quiescence or suppression. The medical coma is often required for several days. A more rational approach would be to implement a brain-machine interface (BMI) that monitors the EEG and adjusts the anesthetic infusion rate in real time to maintain the specified target level of burst suppression. We used a stochastic control framework to develop a BMI to control medically-induced coma in a rodent model. The BMI controlled an EEG-guided closed-loop infusion of the anesthetic propofol to maintain precisely specified dynamic target levels of burst suppression. We used as the control signal the burst suppression probability (BSP), the brain's instantaneous probability of being in the suppressed state. We characterized the EEG response to propofol using a two-dimensional linear compartment model and estimated the model parameters specific to each animal prior to initiating control. We derived a recursive Bayesian binary filter algorithm to compute the BSP from the EEG and controllers using a linear-quadratic-regulator and a model-predictive control strategy. Both controllers used the estimated BSP as feedback. The BMI accurately controlled burst suppression in individual rodents across dynamic target trajectories, and enabled prompt transitions between target levels while avoiding both undershoot and overshoot. The median performance error for the BMI was 3.6%, the median bias was -1.4% and the overall posterior probability of reliable control was 1 (95% Bayesian credibility interval of [0.87, 1.0]). A BMI can maintain reliable and accurate real-time control of medically-induced coma in a rodent model suggesting this strategy could be applied in patient care.

## Author Summary

Brain-machine interfaces (BMI) for closed-loop control of anesthesia have the potential to enable fully automated and precise control of brain states in patients requiring anesthesia care. Medically-induced coma is one such drug-induced state in which the brain is profoundly inactivated and unconscious and the electroencephalogram (EEG) pattern consists of bursts of electrical activity alternating with periods of suppression, termed burst suppression. Medical coma is induced to treat refractory intracranial hypertension and uncontrollable seizures. The state of coma is often required for days, making accurate manual control infeasible. We develop a BMI that can automatically and precisely control the level of burst suppression in real time in individual rodents. The BMI consists of novel estimation and control algorithms that take as input the EEG activity, estimate the burst suppression level based on this activity, and use this estimate as feedback to control the drug infusion rate in real time. The BMI maintains precise control and promptly changes the level of burst suppression while avoiding overshoot or undershoot. Our work demonstrates the feasibility of automatic reliable and accurate control of medical coma that can provide considerable therapeutic benefits.

## Introduction

Medically-induced coma (also referred to as medical coma) is a drug-induced state of profound brain inactivation and unconsciousness used to treat refractory intracranial hypertension and status epilepticus, i.e., epilepsy that is refractory to standard medical therapies [1]–[3]. Following a traumatic brain injury, an anesthetic drug such as a barbiturate or propofol, is administered continuously to provide brain protection by decreasing the cerebral metabolism and blood flow, and thereby, intracranial hypertension [2]. In the treatment of status epilepticus the anesthetic is administered to directly inhibit activity in the seizure foci [3]. For treating both refractory intracranial hypertension and status epilepticus, the state of medical coma is achieved by continually monitoring the patient's brain activity with the electroencephalogram (EEG) and titrating the anesthetic drug infusion rate to maintain a specified level of burst suppression. Burst suppression is an EEG pattern characterized by intervals of electrical bursts that alternate with isoelectric or quiescent intervals termed suppressions [4], [5] and is an EEG marker of profound brain inactivation. In most cases, once burst suppression is achieved, it can be controlled by decreasing or increasing the infusion rate of the anesthetic to decrease or increase the suppression level.

No guidelines have been set to define what level of burst suppression should be achieved to maintain a medical coma [3]. A common practice is for the intensive care unit team to agree upon a target level of burst suppression, monitor continually the EEG and adjust manually the infusion rate of the anesthetic to maintain the target level. In most cases, the medical coma is required for at least 24 hours and frequently longer. It is not realistic to expect intensive care unit staff to maintain reliable and accurate control of a patient's brain state for such a long period by manually changing the infusion rate of the anesthetic in response to changes in the EEG observed in the bedside monitor. A more rational approach would be to define numerically a target level of burst suppression and implement a computer controlled system or a brain-machine interface (BMI) that monitors the actual level of burst suppression based on the brain's EEG activity and adjusts the rate of the anesthetic infusion pump as needed in real time to maintain the target level.

When used to control the delivery of anesthetic drugs, BMIs are often termed closed loop anesthetic delivery (CLAD) systems. During the last 60 years considerable work has been done on the development of CLAD systems for maintenance of general anesthesia and sedation (see Discussion). Interest in CLAD systems has grown driven by attempts to design more efficient, cost-effective ways to administer anesthesia care. To date, no CLAD system has been developed to manage medical coma. Systems to control burst suppression have only been studied in rodent models. Vijn and Sneyd implemented a CLAD system in a rodent model to establish a paradigm for testing new anesthetics [6]. Cotten and colleagues used the Vijn and Sneyd paradigm to study new etomidate-derived anesthetics in a rodent model [7]. Both studies reported average control results rather than results for individual animals and controlled constant target levels of burst suppression rather than time-varying target levels. Here we present a BMI using a stochastic control framework for control of time-varying burst suppression target trajectories in individual rodents. Our study uses a rodent model to establish the feasibility of automatic control of burst suppression as a way to eventually achieve real-time control of medical coma for therapeutic purposes in humans. We show that for individual rodents the BMI enables accurate maintenance of multiple desired target levels within the same experimental session, enables prompt transitions between target levels without overshoot or undershoot, and allows specific constraints to be formally imposed over the infusion rates or the vital states (see Discussion).

The presented BMI applies an EEG-guided, closed-loop infusion of propofol to control the level of burst suppression in medically-induced coma in a rodent model using a stochastic control framework. In this framework, we use the concept of the burst suppression probability (BSP) to define the brain's instantaneous probability of being in the suppressed state and quantify the burst suppression level. We use a two-dimensional linear compartment model to characterize the effect of propofol on the EEG. For each animal, we estimate the parameters of the compartment model by nonlinear least-squares in an experiment prior to initiating control. The BMI consists of two main components: an estimator and a controller. We derive a two-dimensional state-space algorithm to estimate the BSP in real time from the EEG thresholded and segmented into a binary time-series. Taking the BSP estimate as the control signal, we derive controllers using both a linear-quadratic-regulator (LQR) and a model predictive control strategy. We first verify the performance of the developed stochastic control framework in a simulation study based on the model parameters estimated from the actual experimental data. We then illustrate the application of our BMI system by demonstrating its ability to maintain precise control of time-varying target levels of burst suppression and to promptly transition between changing target levels without overshoot or undershoot in individual rodents.

## Materials and Methods

### Animal Care and Use and Ethics Statement

Animal studies were approved by the Subcommittee on Research Animal Care, Massachusetts General Hospital, Boston, Massachusetts, which serves as our Institutional Animal Care and Use Committee. Animals were kept on a standard day-night cycle (lights on at 7:00 AM, and off at 7:00 PM), and all experiments were performed during the day.

### BMI Design

#### Overview

**Figure 1:**
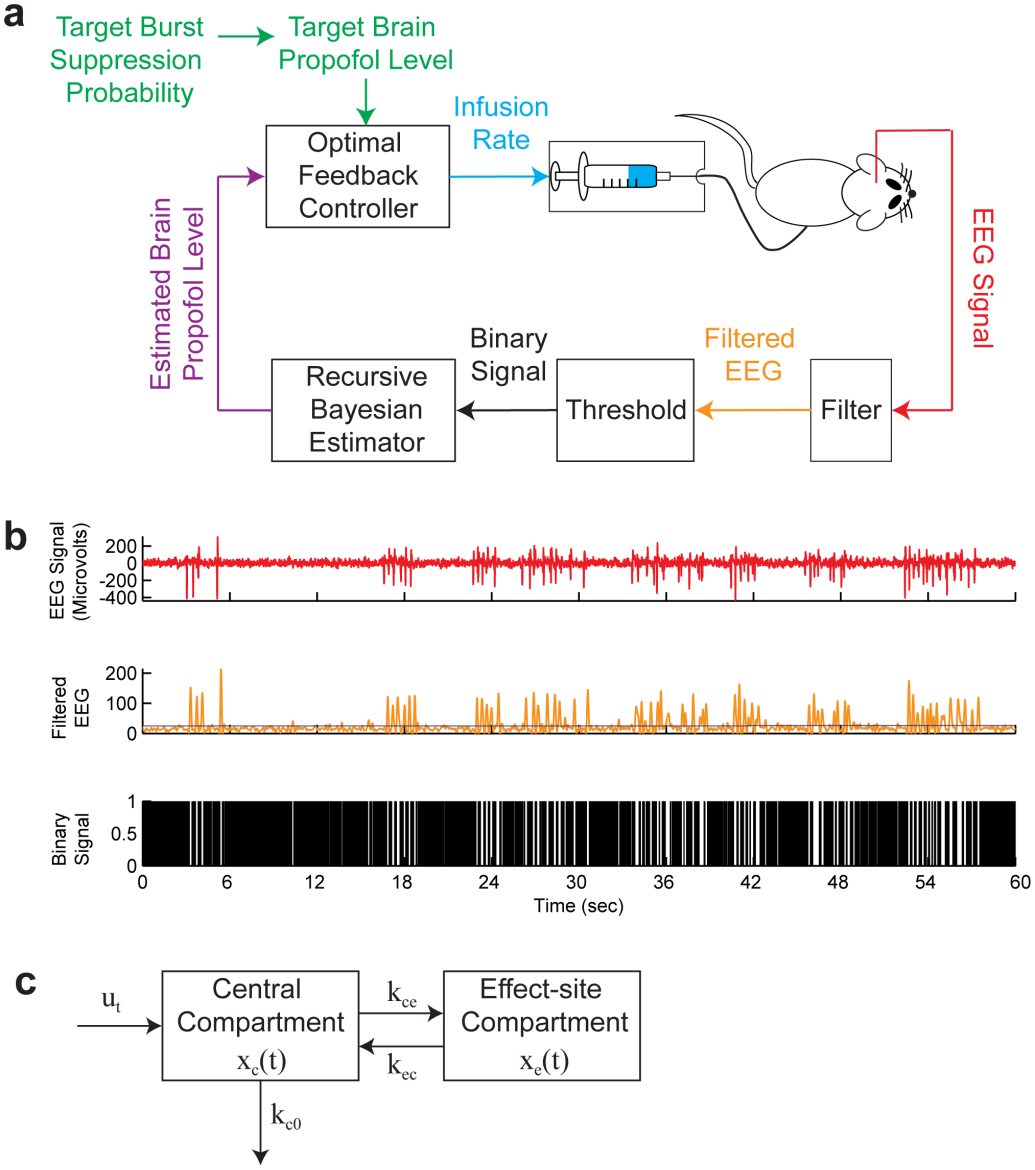
The BMI system. (a) The BMI records the EEG, segments the EEG into a binary time-series by filtering and thresholding, estimates the BSP or equivalently the effect-site concentration level based on the binary-time series, and then uses this estimate as feedback to control the drug infusion rate. (b) A sample burst suppression EEG trace. Top panel shows the EEG signal, middle panel shows the corresponding filtered EEG magnitude signal (orange) and the threshold (blue) used to detect the burst suppression events, and bottom panel shows the corresponding binary time-series with black indicating the suppression and white indicating the burst events. (c) The two-compartmental model used by the BMI to characterize the effect of propofol on the EEG.

**Figure 2:**
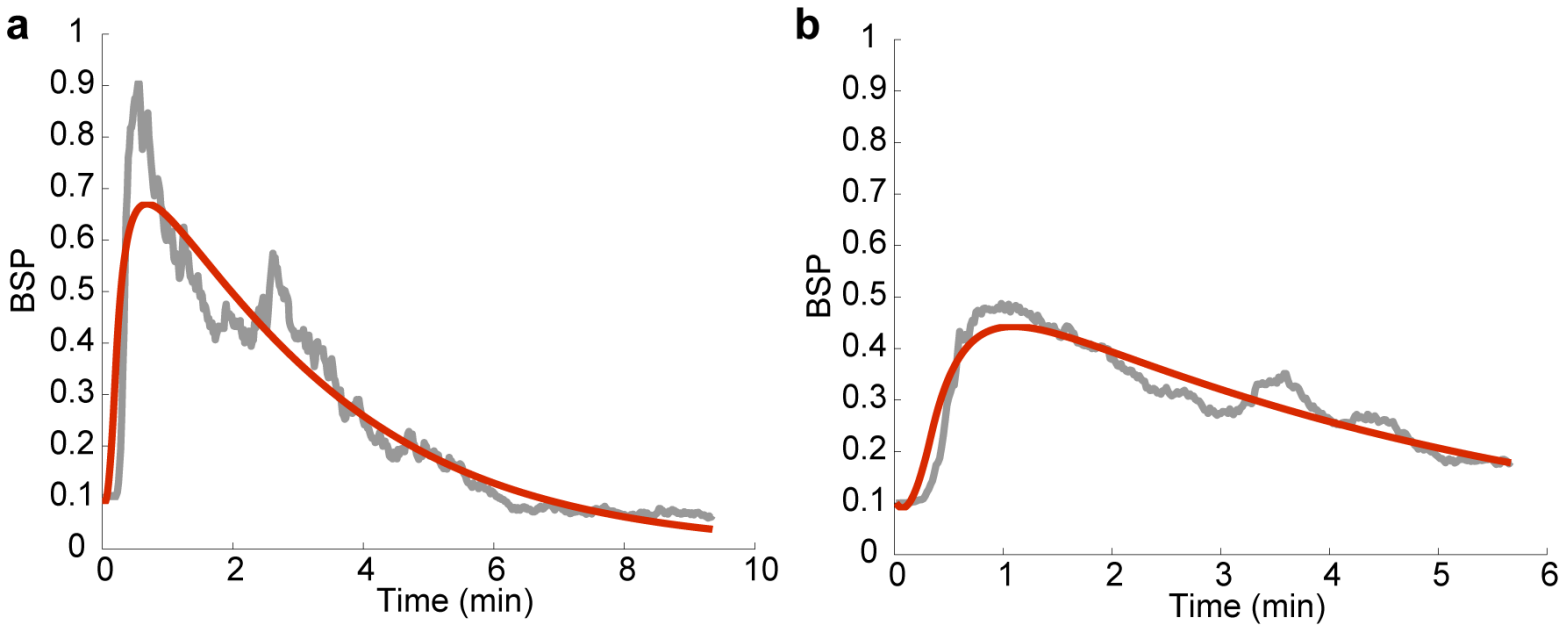
System identification. (a) and (b) show two sample fitted system responses. The measured BSP trace in response to a preliminary bolus of propofol is shown in grey and the response of the second-order system model in (2) fitted using nonlinear least-squares is shown in red.

We use a stochastic optimal control paradigm to design a real-time BMI to control medical coma using burst suppression (Figure 1a). As our measure of the burst suppression level, we use the burst suppression probability (BSP), a number between 0 and 1, which defines the instantaneous probability of the EEG being suppressed. The BSP is computed in one-second intervals in real time by filtering and thresholding the EEG to convert it into binary observations (Figure 1b). To estimate the BSP from the binary observations we first formulate a two-dimensional compartment model that relates the BSP to the concentrations of the anesthetic in the central compartment and the effect site compartment (Figure 1c). We next estimate the parameters of the compartment model based on the EEG observations recorded in a systems identification experiment conducted prior to initiating real-time control (Figure 2). We carry out our stochastic control framework by developing from the two-dimensional compartment model a recursive Bayesian estimator of the concentration states and consequently of the BSP from the binary observations in real time (14)–(17). We develop a LQR controller that takes the concentration estimates as feedback and determines the drug infusion rate in real time (25). In addition to the LQR control strategy, we also implement a model predictive controller (MPC) that allows us to explicitly impose constraints on the anesthetic infusion rates (27). We present the mathematical details of the system identification, formulation of the Bayesian estimator and the two controllers for the interested readers below. These mathematical details in this subsection are not necessary to follow the remainder of the paper beginning with the Results.

#### Problem formulation

Our goal is to control the anesthetic state of the brain in burst suppression, which depends on the effect-site (i.e., brain) drug concentration. The burst suppression state or the effect-site concentration, however, are not directly observable. What we observe is the EEG signal, a stochastic process that depends on the burst suppression state. To design the closed-loop BMI, we present a certainty-equivalent optimal feedback control approach [8] by deriving an estimator for the burst suppression state based on the EEG observations and designing an optimal feedback controller that takes this estimate as a feedback signal to control the drug infusion rate in real time (Figure 1a).

As our measure of the burst suppression state, we use the burst suppression probability (BSP) by filtering and thresholding the EEG signal in small intervals to identify the activity in each interval as a burst or a suppression event (see Experimental Procedure; Figure 1b). BSP is then defined as the brain's instantaneous probability of being in the suppressed state at a given time interval. We denote the BSP at time 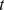 by 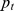. The BSP is in turn related to the effect-site drug concentration. Since higher levels of effect-site concentration should result in higher levels of BSP and since BSP should be a number between 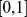, in this work we relate the BSP to a measure of the effect-site concentration, 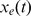, using a hyperbolic transform
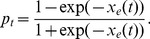
(1)Hence our goal is to control the BSP, or equivalently to control this measure of effect-site concentration, 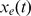.

To develop the estimator and the controller, we construct a state model for the drug concentration state that describes its dynamics in response to propofol infusion. Pharmacokinetic models characterize the dynamics of a drug's absorption, distribution, and elimination in the body (e.g. [9], [10]). We adapt a simplified two-compartment linear pharmacokinetic model [11] to describe the anesthetic drug's dynamics in burst suppression. In this model, one compartment represents the central plasma and the other compartment represents the effect-site or brain (Figure 1c). The anesthetic drug enters the body and is eliminated from the body through the central compartment, and can flow in both directions between the two compartments. In the Results section we show that this model is sufficient to achieve reliable and accurate control of burst suppression.

Given the two compartments in the model, the concentration state is two-dimensional and is denoted by 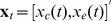, where as before, 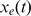 is the brain's anesthetic concentration and 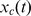 is a measure of the central plasma concentration at time 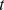. Denoting the sequence of drug infusion rates by 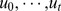, the sequence of anesthetic concentration states in the two-compartment model, 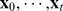, are generated according to the linear dynamical system
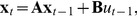
(2)where
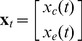
(3)

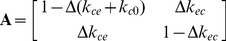
(4)

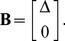
(5)Here 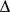 is the discretization time step, and 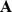 (or equivalently 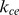, 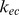, and 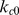) are parameters of the two-compartment model that we need to estimate (Figure 1c). We estimate this model for each animal from the EEG data prior to initiating real-time control as discussed in detail in the System Identification section.

We first derive a recursive Bayesian estimator of the burst suppression level from the EEG thresholded and segmented into a binary time-series. We then derive an optimal feedback-controller that uses this estimate as a feedback signal to decide on the drug infusion rate in real time.

#### Recursive Bayesian estimator

We now develop a recursive Bayesian estimator for the drug concentrations and consequently for the BSP based on the binary observations of the thresholded EEG signal. Since the drug concentration state, 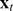, is a positive variable, we estimate its logarithm, 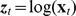, from the EEG signal instead.

A recursive Bayesian estimator consists of two probabilistic models: the prior model on the time sequence of the concentration states, and the observation model relating the EEG signal to these states [12], [13]. Using the two compartment model in (2), we write the prior model for 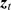 as
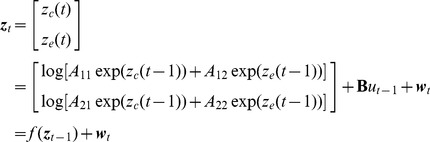
(6)where 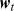 is a zero-mean white Gaussian noise with covariance matrix 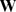 and summarizes the uncertainties in the state model, and 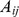 is the 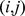
th component of 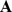. At time 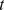, 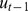 is the known drug infusion rate used by the BMI in the previous time step. Note that our prior state model is nonlinear.

The observation in the estimator is the binary time-series of the burst suppression events obtained by thresholding the EEG (see Experimental Procedure; Figure 1b). To construct the observation model, we assume that in each time interval 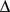 there can be at most 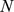 suppression events and that the number of such suppression events is binomially distributed with burst suppression probability 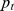. Denoting the number of suppression events by 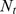, the observation model is given by
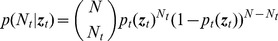
(7)where we have indicated the dependence of the BSP, 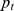, on the states, 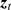, explicitly.

Using the prior and observation models in (6) and (7), we now derive the recursive Bayesian estimator. The estimator's goal is to causally and recursively find the minimum mean-square error (MMSE) estimate of the state 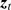 at each time step, which is given by the mean of the posterior density at that time step, 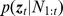. To derive the recursions and denoting the sequence of suppression counts by 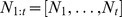, using the Bayes rule we can write the posterior as
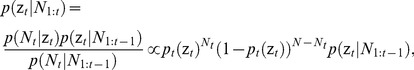
(8)which states the posterior density as a function of prediction density, 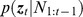. Note that we have used 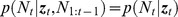, since we assume that the observations of the EEG at a given time step only depend on the concentration states at that time step and hence are conditionally independent of the previous EEG observations. Using the Chapman-Kolmogorov equation, we can in turn write the prediction density as
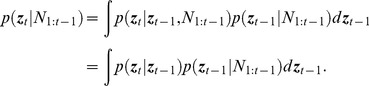
(9)Here we have used the conditional independence, 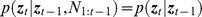, which comes from the prior model in (6). Now the second term inside the integral is just the posterior density from the previous time step. Hence substituting (9) into (8) generates the recursion. The exact expression in (8) is in general complicated and not easy to find analytically. In the special case when both the prior and observation models are linear and Gaussian, these recursions have exact analytical solutions and result in the celebrated Kalman filter. In our case, however, first, the prior model is nonlinear and second, the observation model is not Gaussian but binomial. Hence we make two approximations at every time step to compute the recursions. First, similar to the case of the extended Kalman filter, we make a linear approximation to the prior model at each time step. Second, we make a Gaussian approximation to the posterior at each time step.

We denote the mean of the posterior, i.e., 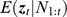, by 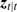 and its covariance matrix by 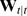. Similarly, we denote the mean of the one step prediction density, 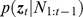, by 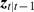 and its covariance matrix by 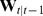. As the first approximation, we linearize the prior model in (6) around the posterior mean, 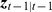. Doing so we have
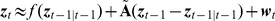
(10)where
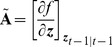
(11)

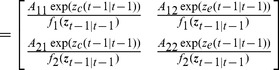
(12)with 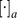 denoting the evaluation of the inside expression at value 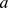 and with
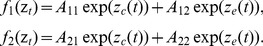
(13)


As the second approximation, we make a Gaussian approximation to the posterior density. Doing so, from (9) the prediction density will be approximately Gaussian since 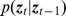 is approximately Gaussian from (10). Using (10) we can find the prediction mean and covariance as
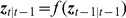
(14)

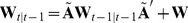
(15)This is the prediction step of the estimator. Now making the Gaussian approximation we get the update step (see Supporting Text S1 for details)
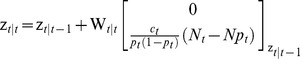
(16)

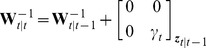
(17)where again 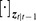 indicates the evaluation of the inside expression at 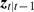 and
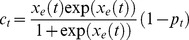
(18)

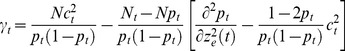
(19)with
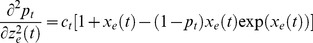
(20)


Hence (14)–(17) give the estimator recursions. The estimator finds the MMSE estimate of the state or equivalently the posterior mean at time 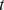 in two steps: first, before data 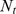 is observed, it uses the prior model in (6) to make a prediction on the state, i.e., find 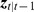 given 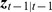 and 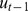
—this is the prediction step in (14) and (15). Once data 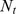 is observed, it combines the observation model in (7) with the prediction density to find the posterior mean 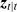
—this is the update step in (16) and (17). Consequently since 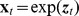, we find the concentration state estimate as 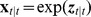, and since the BSP is related to 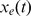 by a hyperbolic transform in (1), we estimate it as 
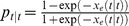
.

#### Optimal feedback-controller

The recursive Bayesian estimator derived above provides us with a real-time estimate of the concentration states at each time step. We now design a real-time optimal feedback-controller that takes as feedback this state estimate and decides on the sequence of drug infusion rates 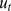 to control the BSP. To find 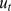 in the optimal control framework, we need to quantify the goal of the controller in a cost function that will then be minimized by selecting the optimal 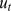. For the linear state model in (2), if we pick the cost function as a quadratic function of the state and control signals given by
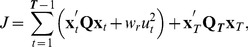
(21)where 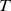 is the time duration of anesthesia, 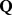 and 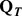 are positive semidefinite and 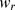 is positive, then the optimal control signal at any time, 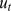, is simply a linear feedback of the state at that time given by [8]

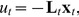
(22)where the feedback matrices, 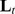, can be found recursively and offline [8]. This is the linear-quadratic-regulator (LQR) solution. Moreover, when the state model 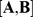 is controllable (as is the case in our problem using the experimental fits; see Results), there exists a steady-state solution, 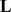, for the feedback matrix in (22). This steady-state feedback matrix is the solution to the discrete form of the famous algebraic Riccati equation given by [8]

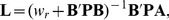
(23)where
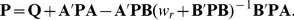
(24)


In the general LQR formulation above, however, the goal is to derive the states close to *zero*—while limiting the total amount of control—as evident from the cost function in (21). In the control of burst suppression, our goal is to achieve a desired *non-zero* target BSP level, 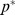, or equivalently to take the effect-site concentration state close to a non-zero target level 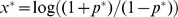, using as little drug as possible. Hence to find the solution, we additionally shift the origin of the state-space to 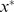 [14]. This way, the control goal is equivalent to deriving the shifted state variable close to zero, as in the classical LQR formulation. We show in the Supporting Text S1 that, in our problem, it is possible to shift the origin and the optimal drug infusion rate is in turn given by
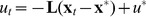
(25)where
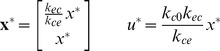
(26)The value of 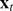 at each time step is provided by the estimator.

Note that the LQR formulation does not impose any constraints, such as positivity of the control variables. In practice we can impose these constraints by bounding the LQR control solution in (25) appropriately (for example if the solution is negative, use zero instead). Another way to solve optimal control problems with constraints is to use a model predictive control approach as we develop next.

#### Model predictive controller

One approach to solve the optimal control problem while explicitly imposing constraints on the state and control variables is to use a model predictive controller that approximately solves the constrained optimal control problem at each time step 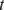 [8]. In model predictive control, at every time step 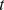 we solve the optimization problem
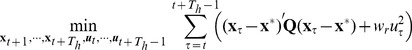
(27)

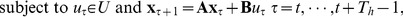
(28)where 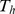 is a finite control horizon, 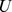 is a convex set of permissible control values, and 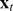 is the input to the optimization problem at time 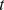 and is given by the estimator (i.e., 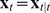). If needed, we can also add constraints on the permissible state variables in the optimization problem, i.e., 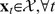. Hence in every time step, we solve one quadratic optimization problem over the state and control variables. Since solving this constrained optimization problem over the entire time course is computationally expensive, smaller time horizons, 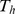, are selected in practice. Solving this optimization problem and denoting the solutions by 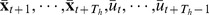, the model predictive controller takes 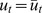 as the drug infusion rate at time 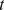. Note that again the optimal control or drug infusion rate is a function of the value of the current state 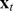, though a complicated function (i.e., 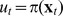). This process gets repeated for every time step.

In our BMI, we implement both the bounded LQR controller and the model predictive controller in which such constraints (such as positivity of drug infusion rates) are explicitly imposed in the formulation. We show that in our problem, in which there are only constraints on the drug infusion rate or equivalently the control variable, the two approaches yield approximately the same infusion rates. However, as we expand on in the Discussion section, the recursive Bayesian estimator combined with the implemented model predictive controller could solve more general problems in which constraints are also required on the state variables and could extend our framework to the joint control of the anesthetic state and other vital states such as blood pressure.

### Experimental Setup and Signal Acquisition

Surface EEG recordings were collected using extradural electroencephalogram electrodes that were surgically implanted at the following 4 stereotactic coordinates relative to lambda: A (Anterior) 0 mm L (Lateral) 0 mm, A6L3, A6L-3, and A10L2 [6], [15], [16]. During implantation, general anesthesia was induced with isoflurane. At the above four stereotactic coordinates, four holes were made using a microdrill (Patterson Dental Supply Inc., Wilmington, MA). An electrode with mounting screw and socket (Plastics One, Roanoke, VA) was screwed into each of these four holes. The sockets were in turn inserted in a pedestal. Dental acrylic cement was used to permanently fix the screws, sockets and pedestal. Recording began after at least 7 days of recovery following implantation.

During the experiment, the potential difference between electrodes A0L0 and A6L3 was recorded and the signal was referenced to A10L2 and recorded using a QP511 Quad AC Amplifier System (Grass Instruments, West Warwick, RI) and a USB-6009 14-bit data acquisition board (National Instruments, Austin, TX). The binary signal was acquired at a sampling rate of 500 Hz and fed into our BMI. Our algorithm was implemented in a simulink-matlab framework on a HP Probook 5430 s laptop. This setup controlled a Physio 22 syringe pump (Harvard Apparatus, Holliston, MA) to deliver the propofol infusion rate. A 24 gauge intravenous catheter was placed in a lateral tail vein during brief general anesthesia with isoflurane (2% to 3%) in oxygen, and then the animal was allowed to fully recover from the isoflurane general anesthetic in room air before the start of the experiment. The temperature of the animal was monitored and maintained in the normothermic range for the duration of the experiment.

### Experimental Procedure

For all experiments, the magnitude of the raw EEG signal was low-pass filtered below 5 Hz and then thresholded to convert it into a binary signal. At the start of an experiment, the threshold level was empirically chosen based on visual inspection of the BSP and the corresponding binary data and based on the values of the filtered EEG over the bursts and suppressions. Figure 1b shows the burst-suppression raw EEG, filtered EEG and threshold, and the resulting binary signal. The segmentation algorithm was run in real time. Several preliminary boluses of propofol were administered to each rat and the obtained BSP traces were used for system identification in each animal (see System Identification section below). The experiment was then conducted by giving the rat a manual propofol bolus to induce a burst suppression state, and the real-time BMI control experiment started once the BSP dropped to a level of 0.1–0.3. In the real-time BMI experiments, the goal was to acquire, maintain, and transition between three target BSP levels (low, medium, high). The order of the target levels was randomized. Each real-time BMI control experiment was conducted for an average of 62 min. Three rats were available for the experiments, weighing 366, 391, and 422 gr respectively. Each rat was used for two real-time experiments, resulting in six real-time experiments.

### System Identification

Our system identification procedure is conducted prior to real-time BMI control for each animal in a preliminary experiment and consists of two steps. First, a BSP signal is estimated from the binary thresholded EEG trace using a special case of our recursive Bayesian estimator in which we take the state to be the scalar variable 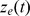. Hence the corresponding state model in the estimator imposes a smoothness constraint on 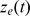 using a first-order linear Gaussian process [17]. Specifically, we use a special case of (10) as 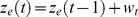. Second, the corresponding BSP estimate 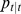 is fitted using a non-linear least-squares procedure to minimize the sum-squared-error between the model predicted BSP and the estimated BSP. The system parameters are thus the solution to
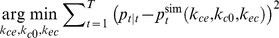
(29)where 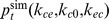 is the model predicted BSP given the values for the system parameters (see Results and Figure 2).

### Experimental Performance Metrics

To characterize the performance of the BMI at steady state, we compute the error between the target BSP at each time, 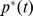, and the controlled BSP, 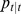, as
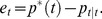
(30)


We use the error to calculate multiple standard metrics [18] of performance. These metrics are the median absolute deviation (MAD)
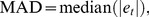
(31)the median prediction error (MDPE)
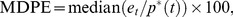
(32)and the median absolute performance error (MDAPE)
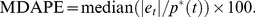
(33)The MDPE is a measure of bias at steady state and the MDAPE is a measure of normalized error. We compute these metrics for low, medium, and high target BSP levels and across all levels for each experiment. The median is computed across data points at steady state. Finally we compute the median of all these measures across all experiments to quantify the overall performance of the BMI.

To characterize the performance of the BMI in transitioning between target BSP levels, we calculate the rise time for an upward transition and the fall time for a downward transition. These are defined as the time it takes, once the target is changed, for the BSP to reach within 0.05 BSP units of the new target BSP. We then find the rate of BSP change defined as
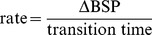
(34)and calculate the median of this rate across all upward transitions and also across all downward transitions.

### Bayesian Analysis of BMI Performance

In addition to calculating the steady-state error metrics above for the low, medium, and high levels in each experiment, across all levels for each experiment, and across all experiments (Table 1), we also performed specific statistical assessments based on the error distribution at each level to examine the reliability of the BMI overall [19]. In particular, we considered the BMI to be reliable at each level if its absolute error measure, 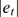, was lower than a specified value with high probability. Experimentally we found that the absolute error at any time step in our BMI system was almost always below 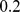. Therefore we considered the BMI to be reliable at a given level if its absolute error at that level was less than 0.15 with probability ≥0.95 and *highly* reliable if the absolute error at each level was less than 0.10 with probability ≥0.95. This is equivalent to the 95th percentile of the absolute error distribution at a given level being less than 0.15 and 0.10, respectively. Hence we can compute from the absolute error distribution at each level the 95th percentile and consequently identify the BMI performance at each level as reliable or not.

**Table 1:** Performance metrics across experiments.

Experiment #	1	2	3	4	5	6	Median
Low Level							
Median Abs. Dev.	0.027	0.043	0.017	0.032	0.034	0.031	**0.032**
Median Abs. Perf. Error	5.43	10.72	4.90	11.44	8.47	6.18	**7.32**
Median Pred. Error	−0.80	10.72	1.79	−11.44	−8.47	−3.04	**−1.92**
Mid Level							
Median Abs. Dev.	0.018	0.016	0.019	0.017	0.052	0.019	**0.019**
Median Abs. Perf. Error	2.62	2.32	3.80	3.35	7.37	2.68	**3.02**
Median Pred. Error	1.75	1.00	−0.40	−1.45	−7.37	−0.06	**−0.23**
High Level							
Median Abs. Dev.	0.012	0.016	0.031	0.038	0.043	0.017	**0.024**
Median Abs. Perf. Error	1.35	1.75	3.69	4.35	4.81	1.87	**2.78**
Median Pred. Error	−0.81	−1.73	−3.69	−4.35	−4.81	−1.84	**−2.77**
All Levels							
Median Abs. Dev.	0.019	0.022	0.021	0.032	0.041	0.022	**0.022**
Median Abs. Perf. Error	2.82	3.01	4.14	4.98	5.61	3.07	**3.61**
Median Pred. Error	−0.13	0.99	−1.24	−4.87	−5.60	−1.63	**−1.44**

After evaluating the reliability of the BMI at each level separately, we use a Bayesian analysis to identify the reliability of the BMI across all levels. To do so, we combine the results of the reliability assessments across all levels to compute an overall assessment of reliability for the experiment. In our experiments, we tested the BMI over 20 levels with the time duration at each level between target transitions being 18.6 minutes on average. For the purpose of steady-state error calculation, we remove 5 minutes of data after an upward transition and 7 minutes of data after a downward transition to ensure that the system is at steady-state and to ensure approximate independence between levels. The independence assumption between levels is justified because if we assume even a high first-order serial correlation of 0.98 between adjacent data points separated by one second and we allow between 5 to 7 minutes for the transition between levels before making the steady-state error calculations, then the maximum correlation between the closest two points in immediately adjacent levels is between 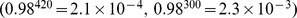, where 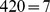 min 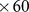 data point per minute and 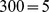 minutes 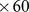 data points per minute. Because these maximum correlations are effectively 0, assuming independence between levels is highly reasonable (we acknowledge that lack of correlation is not equivalent to independence). Hence the data between levels within animals are approximately independent so that the 20 levels serve as the unit of analysis in the overall assessments of reliability.

Denoting the probability that the BMI system is reliable at any level by 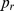, the total number of reliably controlled levels, 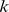, is binomially distributed with success probability 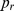 out of 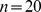 independent levels. The number of successes 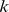 is in turn equal to the number of levels for which the BMI is identified as reliable as described above. Given the binomial likelihood and taking the prior distribution for 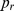 to be the uniform distribution on the interval (0, 1), it follows that the posterior distribution for 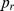 is the beta distribution with parameters 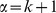 and 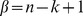 [15], [20]. We thus estimate 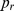 as the mode of this beta distribution and consider the BMI system reliable overall if the leftmost point of the 95% credibility interval for 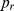 is greater than 0.

## Results

To test our closed-loop BMI system for control of medical coma, we perform both simulation-based verification as well as real-time *in vivo* experiments in rats. In both cases, we implement the recursive Bayesian estimator combined with both the bounded LQR controller as well as the model predictive controller. Using both validation methods, we show that the closed-loop BMI system can accurately control time-varying target levels of burst suppression in real time.

### System Identification

For each experiment, we first performed the system identification step for each animal using the scalar filtering and the nonlinear least-squares model fitting (see Materials and Methods). Figure 2 shows two sample BSP traces in response to boluses of propofol administered in preliminary experiments prior to BMI control, and the fitted system response of the second-order system in (2). The estimated parameters for Figures 2a and 2b are 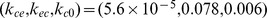 and 
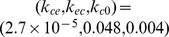
, respectively. Once the system model was fitted, the real-time BMI control experiments were conducted. We use the fitted system model in Figure 2b for our simulation-based verification below.

### Simulations

We first perform a set of simulations to verify the performance of the closed-loop BMI system. In our simulations, we assume that the anesthesia drug delivery period is a total of 45 minutes and that the goal is to keep the BSP at three desired target levels, 0.4, 0.7, 0.9, each for 15 minutes. We simulate all 6 possible order permutations of these levels. To run the simulations, we use the estimated system model in Figure 2b. Note that all the fitted system models in our experiments were controllable.

To specify the cost function (see (27) and Supporting Text (S.18)), we take 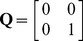. We choose this 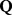 since the main goal is to have the effect-site concentration close to the target value and since the effect-site concentration is the observable through the EEG. The choice of 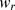 in turn depends on how fast we desire the controller response to be. Smaller values of 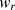 result in faster controller response since the cost on the amount of drug infusion is reduced. Here we pick 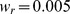 for our desired response.

We take the discretization step to be 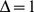 sec. This means that the closed-loop system updates its estimate of the BSP and its drug infusion rate every second. To simulate a trial of the closed-loop controlled system response, at each time 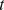 we use 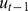 and 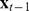 to find 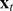 using (2) with initial condition 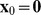, 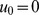. To get the binary output of the thresholded EEG within this time step, we generate a realization of the binomial distribution in (7) with mean 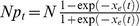 and using a sampling rate of 10 Hz (i.e., taking 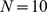). Given this binomial realization, we use the recursive Bayesian estimator to estimate the concentration state 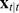, and then use this estimate as feedback in the controller to decide on the infusion rate 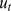.

We impose the constraints on the control (i.e., drug infusion rate) by first finding the unconstrained control solution from (25) and then using the closest value to it in the constrained feasible set 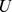. For example, to impose positivity and for negative control solutions we use zero instead. We can similarly do this for constraints on the maximum drug infusion rate.

**Figure 3:**
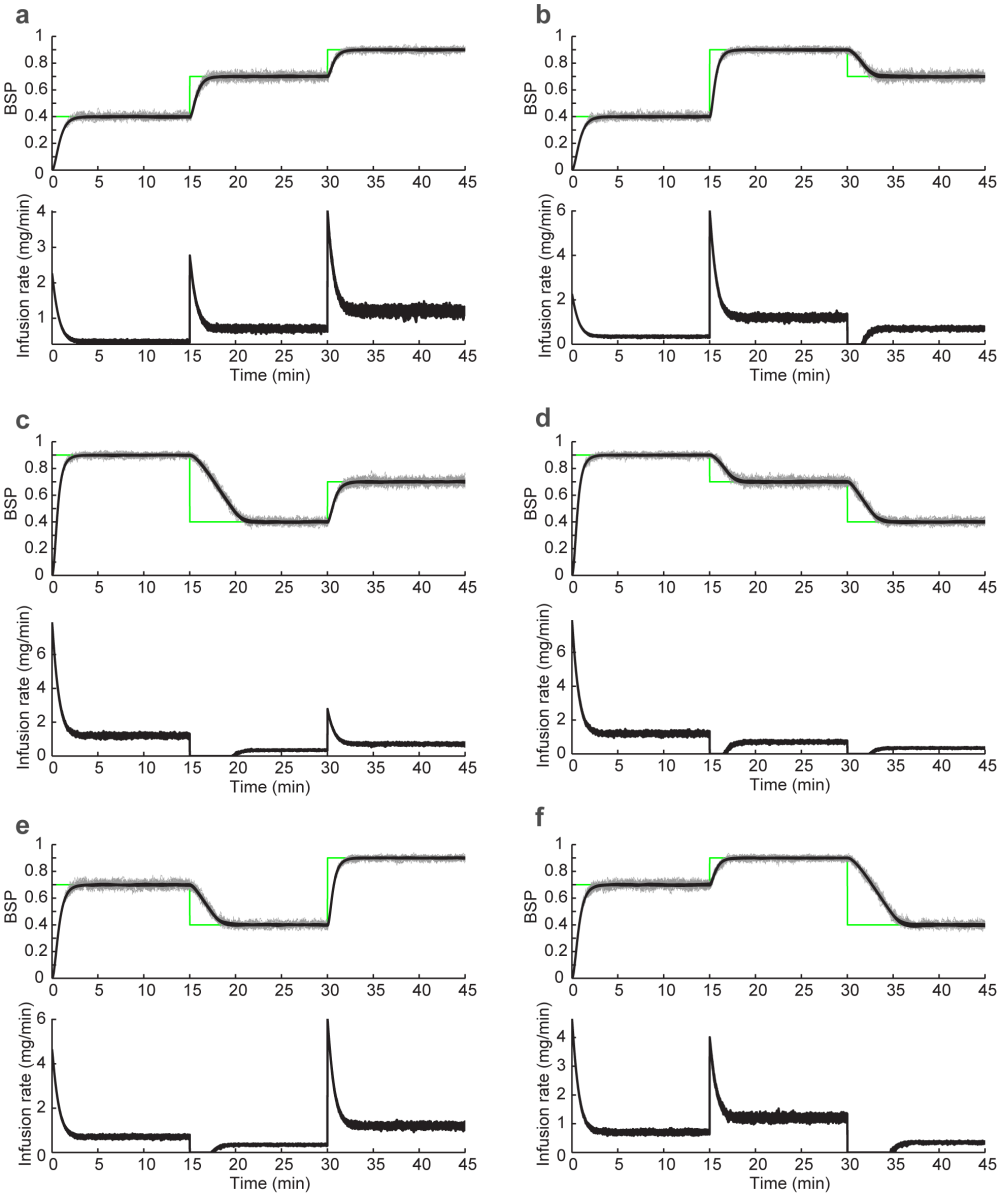
Simulated closed-loop controlled BSP traces. In each subfigure, the top panel shows the BSP traces and the bottom panel shows the drug infusion rate. In the top panels, sample trials of the closed-loop controlled BSP traces are shown in black and the corresponding estimated BSP traces are shown in grey. The time-varying target BSP level is shown in green. The bottom panel shows the corresponding controller infusion rates. Each subfigure (a–f) corresponds to one possible permutation of the 3 BSP target levels.

Figure 3 shows sample closed-loop controlled BSP traces for each of the 6 possible permutations of the desired target trajectories. Here the only imposed constraint is positivity of the drug infusion rates. In each subfigure, the top panel shows the BSP traces and the bottom panel shows the drug infusion rate. The stochastic control framework can achieve successful control of burst-suppression. The framework is particularly successful in changing the BSP level without overshoot or undershoot.

**Figure 4:**
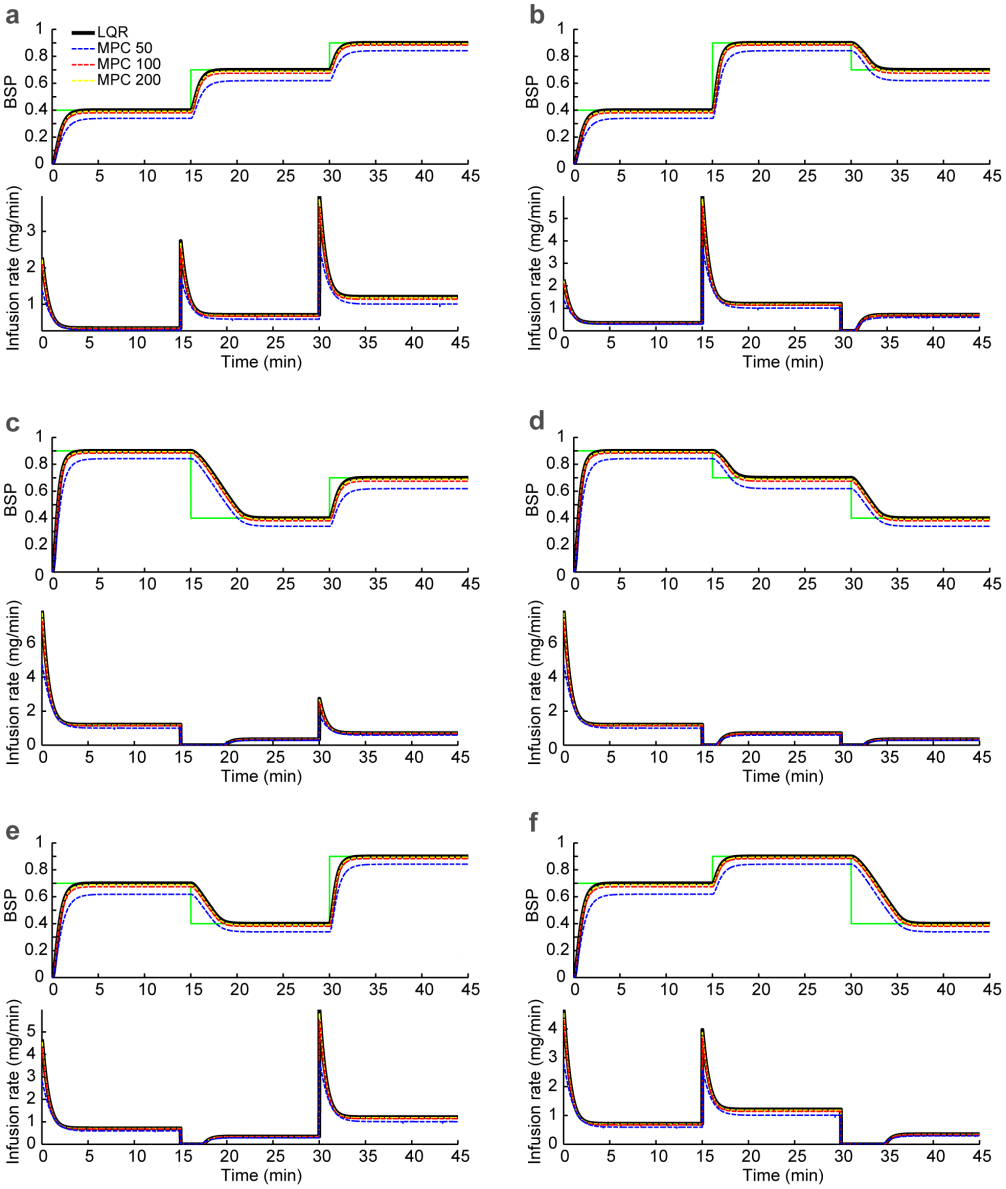
Comparison of the bounded LQR and MPC strategies. In each subfigure, the top panel shows the closed-loop controlled BSP traces using the bounded LQR control strategy and using the MPC strategy with various time horizons, 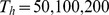 time samples (seconds). The bottom panel shows the corresponding drug infusion rates. The only constraint imposed here is non-negativity of the drug infusion rate. Each subfigure (a–f) corresponds to one possible permutation of the 3 BSP target levels.

**Figure 5:**
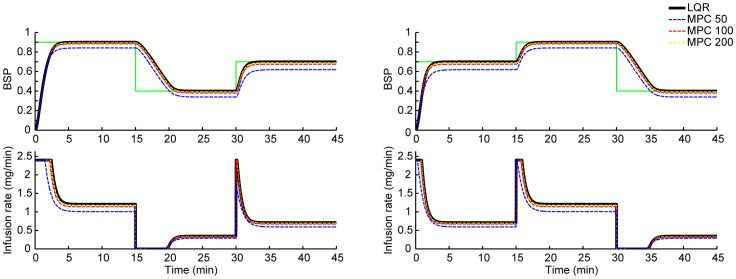
Comparison of the bounded LQR and MPC strategies with upper-bound constraints on the drug infusion rates. In each subfigure, the top panel shows the closed-loop controlled BSP traces using the bounded LQR control strategy and using the MPC strategy with various time horizons, 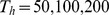 time samples (seconds). The bottom panel shows the corresponding drug infusion rates. In addition to being non-negative, here the drug infusion rate is required to be less than 2.4 mg/min. Here we have shown two example permutations of the target levels but the bounded LQR and the MPC drug infusion rates converge with increasing 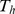 in all cases.

We also tested the model predictive controller with various time horizons, 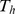. In the model predictive controller, we impose the constraints on the control inputs (i.e., drug infusion rates) explicitly in the formulation and thus find the constrained (approximately) optimal solution. Since our goal is to compare the bounded LQR and MPC control strategies in this set of simulations, we assume that both controllers know the BSP perfectly at each time (i.e., we use the true 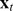 as feedback in the controller). We compare the MPC drug infusion rate with the bounded LQR infusion rate in Figure 4, where the constraint is positivity on the drug infusion rate. As we increase the optimization horizon, the two solutions converge. This shows that, in this problem, solving the unconstrained LQR and then bounding it is approximately optimal. The controlled BSP in Figure 3 is noisier than in Figure 4 because in the former the BSP is estimated from a stochastic binary time-series emulating the segmented EEG (Figure 1b) and in the latter BSP is assumed to be perfectly known to the controllers. We can also show that, similarly, when an upper-bound on the drug infusion rate is desired, the two solutions again converge (Figure 5). It is important to note, however, that in our problem no constraints are placed on the state. Our recursive Bayesian estimator combined with the implemented real-time MPC can extend our framework to solving more complex problems with constraints also on the state variables, such as blood pressure (see Discussion).

Even though simulation-based validations are helpful in assessing the behavior of the BMI, the true test of the BMI is in *in vivo* experiments as we present below.

### BMI for Closed-Loop Control of Medical Coma in Rodents

We implemented our BMI in experiments with rodents and tested it for controlling the level of burst suppression in real time. The BMI used the recursive Bayesian estimator combined with either the bounded LQR controller or the MPC. The BMI in both cases could successfully and accurately control the BSP level in rodents in real time.

**Figure 6:**
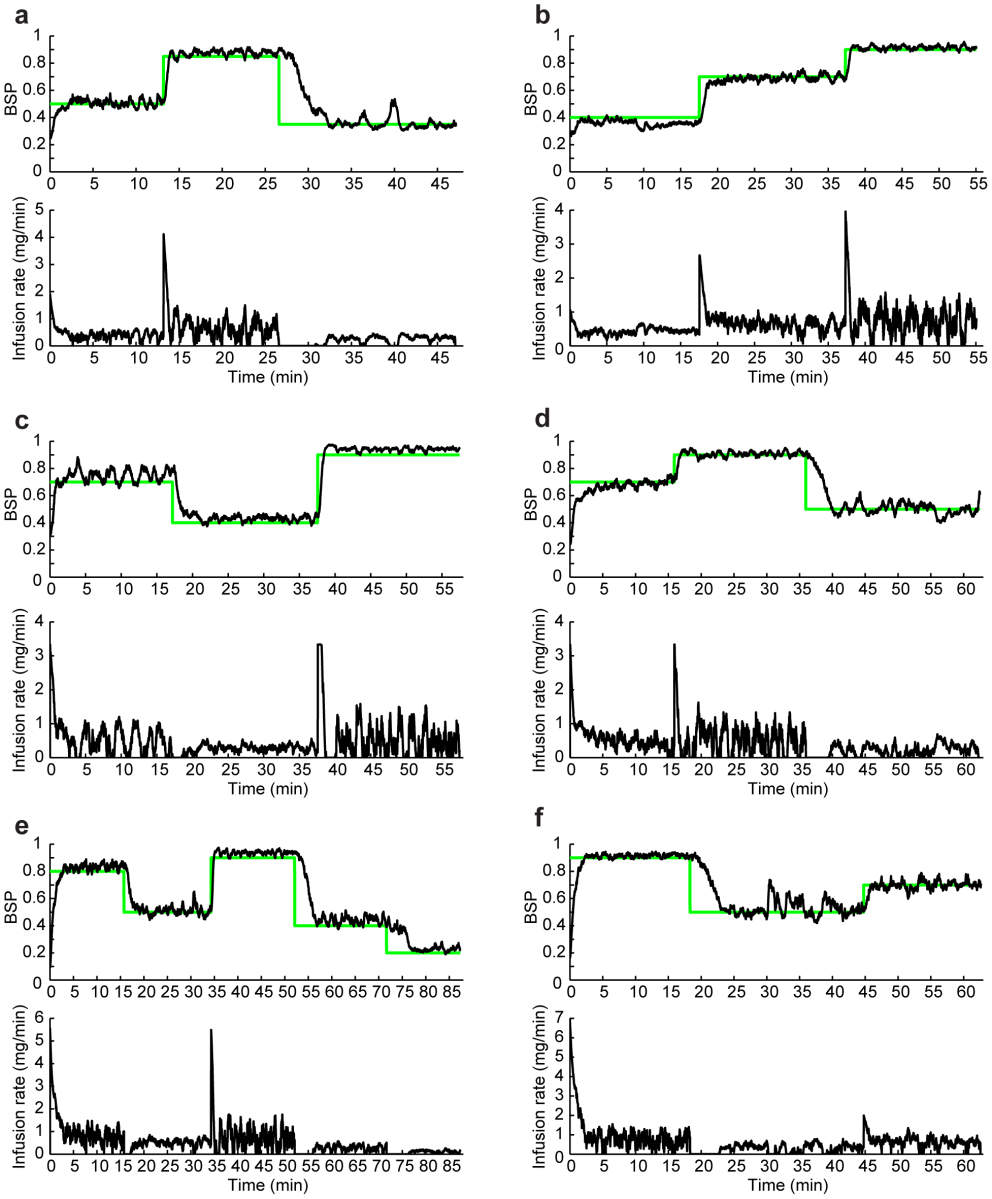
*In vivo* real-time BMI control of burst suppression in individual rodents. In each subfigure, the top panel shows the estimated closed-loop controlled BSP trace (black) and the time-varying target level (green), and the bottom panel shows the corresponding BMI drug infusion rate using the bounded LQR strategy (a–e) and the MPC strategy (f).

The control sessions lasted an average of 62 minutes and consisted of at least 3 target BSP levels, thus requiring at least 3 transitions. Figure 6 shows the BSP and the drug infusion rate in 6 closed-loop BMI sessions that were run in real time in rodents (see also Supporting Figure S1 that shows the evolution of 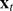 in these experiments). Figure 6a–e were run with the bounded LQR controller and Figure 6f was run with the MPC. All experiments except for the one in Figure 6e consisted of 3 target levels, identified as low, medium, and high levels for the purpose of metric calculation. The experiment in Figure 6e consisted of 5 target BSP levels and hence we identify the lowest two levels as the low level and the highest two levels as the high level to calculate the metrics.

As is evident in Figure 6, the BMI could successfully and promptly transition between levels and accurately maintain the BSP at a desired target level. At steady state, the BMI-controlled BSP closely followed the target BSP level. The real-time variations in the drug infusion rate at higher levels of BSP, e.g., at 0.9, were larger than at the lower levels since larger amounts of propofol are needed to keep the EEG in suppression 90% of the time while allowing for the bursts 10% of the time (this can also be seen from (1) by observing that 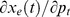 is monotonically increasing with 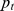). The MDAPE (measure of normalized error) across all experiments for the low, medium, and high target BSP levels was only 7.32%, 3.02%, and 2.78%, respectively. When considering all levels, the MDAPE was only 3.61% (Table 1). Moreover, the deviation between the target BSP level and the BMI-controlled BSP, measured through MAD, was 0.03 BSP units or less for any level. Across all levels the MAD was only 0.02 BSP units (Table 1), a negligible error in practice. Finally, the MDPE was small across all levels. Together, these results demonstrate that the BMI achieved precise control of multiple target burst suppression levels at steady state within the same experimental session.

**Figure 7:**
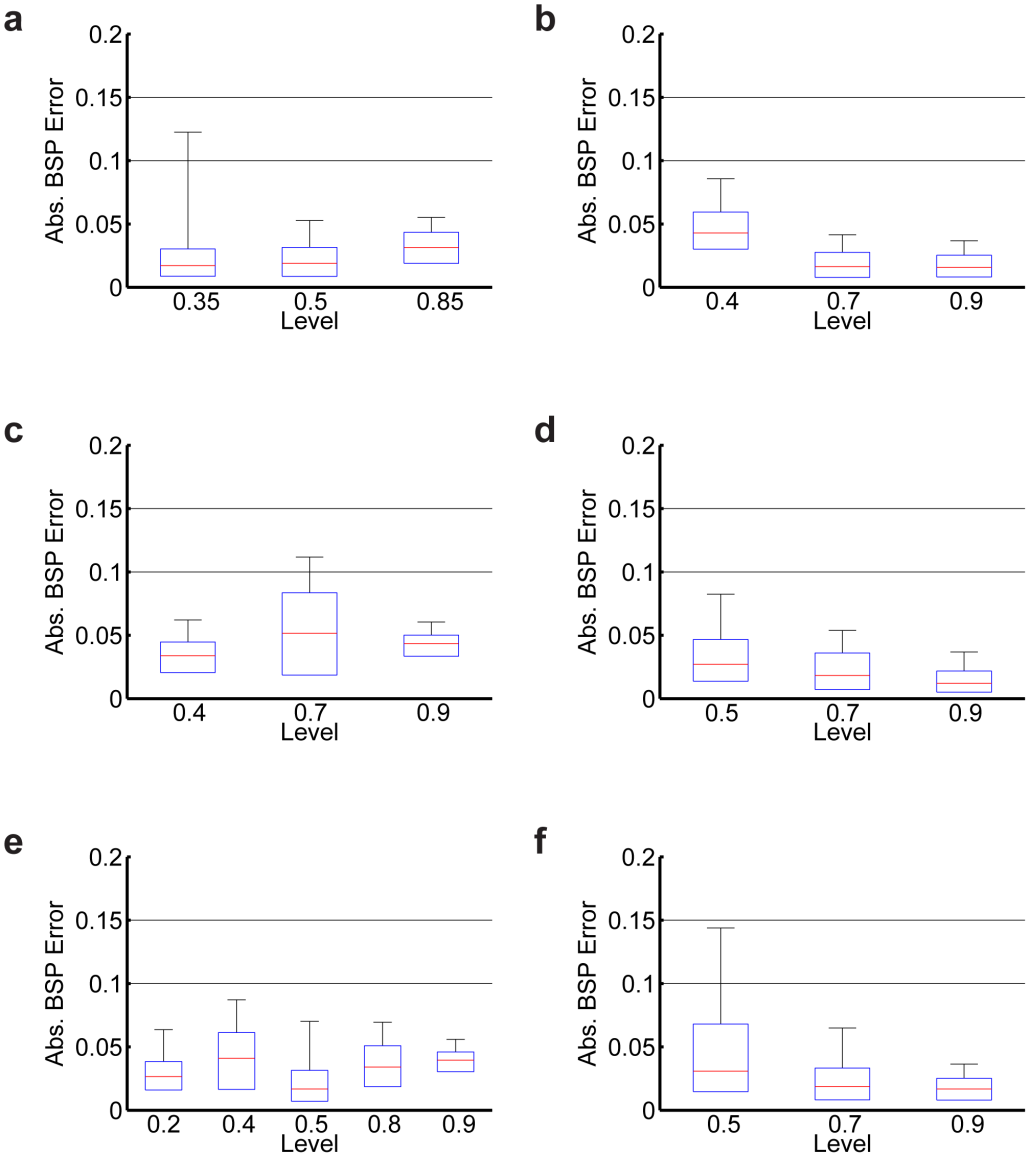
Reliability of the real-time BMI. Each subfigure (a–f) corresponds to one of the six real-time BMI experiments (Figure 6) and shows the modified boxplot summaries for the absolute error distribution at each of the levels used in that experiment. The lower and upper end of the boxes represent the 25th and 75th percentiles of the absolute error distribution and the middle line in each box represents the median. Whiskers represent the 95th percentile of the absolute error distribution at each level. The BMI is reliable (95th percentile of the absolute error <0.15) at all 20 levels. Additionally, the BMI is *highly* reliable (95th percentile of the absolute error <0.1) at 17 of the 20 levels.

We also performed a Bayesian analysis to assess overall reliability of the BMI based on the steady state error distributions at each of the 20 levels used in the experiments (Materials and Methods). The data at different levels within animal are approximately independent so that the 20 levels serve as the unit of analysis in the overall assessment of reliability. The 95th percentile of the absolute error distribution at each of the 20 levels was less than 0.15 giving a mode of the posterior density for 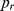 (probability that the BMI is reliable at any level) of 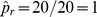 and a 95% credibility (Bayesian confidence) interval for 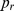 of (0.87 to 1.00) (Figure 7). The lower bound of the 95% credibility interval of 0.87 is well above 0, the point of no control. These findings establish that the system is reliable. In addition, for 17 out of 20 of the levels the 95th percentile of the absolute error distribution was less than 0.1, giving a mode of the posterior density for 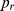 of 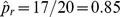 and a 95% credibility interval for 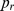 of (0.67 to 1.00) (Figure 7). This finding suggests that furthermore the BMI system meets our definition of being *highly* reliable overall. We therefore conclude that the BMI system is highly reliable for real-time control of medical coma using burst suppression across dynamic targets.

In addition to accurate and reliable control at steady state, the BMI was especially successful in promptly transitioning between target BSP levels. The BMI could increase the level of BSP rapidly, while avoiding overshoot. To increase the BSP, the BMI immediately increased the drug infusion rate once the target was increased, and then gradually reduced the infusion rate until the BSP approached the new target level. The rate at which the BMI increased the BSP level was 0.32 BSP units per minute. The median rise time in our experiments was under a minute (49 seconds).

The BMI was also able to decrease the BSP level without undershoot. To decrease the BSP, the BMI first stopped the drug infusion and then gradually restarted it once the BSP approached the lower target BSP level. The rate at which the BMI could decrease the BSP level was 0.11 BSP units per minute. In decreasing the level of BSP, the time response of the BMI is mainly governed by the clearance rate in the pharmacokinetic model of the rat. Hence although the controller stopped the drug infusion immediately once the target was dropped, it took a few minutes for the BSP to go down to the desired target level. The median fall time in our experiments was 4.45 minutes.

These results thus demonstrate the feasibility of automatic reliable and accurate control of medically-induced coma using a BMI.

## Discussion

To study the feasibility of automating control of medically-induced coma, we developed a BMI to control burst suppression in a rodent model. Our BMI system reliably and accurately controlled burst suppression in individual rodents across dynamic target trajectories. The BMI promptly changed the BSP in response to a change in target level without overshoot or undershoot and accurately maintained a desired target BSP level with a median performance error of 3.6% and a percent bias of -1.4%.

### BMI Development for Control of Anesthesia-Induced Brain States

Our work contributes to the extensive BMI research in anesthesiology aimed at controlling brain states under general anesthesia. This field began in the 1950s [21]–[23] and developed further in the 1980s [24]. BMI systems for control of sedation are now commercially available [25] and have been recently approved for use in the United States. The most commonly used control signal is the Bispectral Index (BIS) [26]–[40]. Other control signals include an auditory evoked potential index [41], the spectrogram median frequency [24], [42], [43], a wavelet-based index [44] and an entropy measure [45]. Both standard and non-standard control paradigms [24], [27], [35], [41], [45] have been used in these systems with the principal objective being control of unconsciousness [24], [26]–[32], . A recent report controlled both antinociception and unconsciousness [45]. Although several criteria have been established for successful control, a criterion used in BIS studies has been maintaining BIS not at a specific value but in the broad range between 40 to 60 [26]–[39]. Vijn and Sneyd [6] and Cotten et al. [7] controlled constant target levels of burst suppression in rodent models and reported average control results over rodents. Schwilden demonstrated control of median frequency in individual human subjects [24]. None of these studies considered control of dynamic time-varying trajectories.

We developed a BMI for real-time control of burst suppression across time-varying target levels in individual rodents using a stochastic control framework. Our stochastic control framework consists of a two-dimensional state estimator and an optimal feedback controller. In our formulation, we assumed a stochastic form of the log transformed version of our system to incorporate both the two-dimensional system model and noise in our estimates and to ensure non-negative concentration estimates (Eqs. (2) and (6)). This model-based two-dimensional state estimator is one major reason that the current BMI largely avoided overshoot and undershoot. By incorporating the two-dimensional stochastic dynamic model and computing both 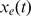 and 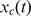 at each update (Eqs. (14)–(17)), the estimator predicted the effect of the real-time drug infusion rate on the BSP. In upward transitions, this avoided underestimating the BSP in response to drug infusion that would result in overestimating the required amount of drug and hence in an overshoot. This similarly prevented undershoot in downward transitions. Our framework is thus analogous to maintaining control in a navigation system by estimating both position and velocity.

In addition to the two-dimensional estimation algorithm, the BMI consists of LQR and MPC controllers. Controllers using MPC and LQR strategies have been used successfully in many applications. We recently demonstrated the success of a LQR paradigm to control a motor neuroprosthetic device using point process observations of spiking activity and a linear Gaussian kinematic state model [46]–[50]. MPC has been widely used in process control and chemical industries [51]–[53] and has been previously applied to closed-loop administration of analgesics [54], for sedation control using the BIS as the control signal [55] and in a simulation study for control of BIS during surgery [40]. The LQR and MPC controllers are both formulated in an optimal feedback control framework [8]. They specify the control objective as a cost function to be minimized by selecting the optimal infusion rates. We can therefore adjust the behavior of these controllers, for example the speed of transitions, by adjusting the penalty on various terms in the cost function. While our LQR implementation imposes constraints on the drug infusion rates by bounding the control solution, our MPC implementation allows us to impose explicitly any required constraints on both the states and the drug infusion rates, such as non-negative or bounded infusion rates, by solving a constrained optimization problem at each time step in real time. For example, if the BMI system always needed to keep the drug infusion rate below a specified maximum level, the MPC controller could impose this explicitly in the solution. Since the only constraints in our problem were on the control variable (i.e., the infusion rate), the LQR and MPC strategies performed similarly (Figure 5). However, the recursive Bayesian estimator combined with the real-time MPC strategy can be used to solve problems that require constraints on the state variables as well. This situation could arise in problems requiring joint control of multiple state variables, such as controlling simultaneously the anesthetic level and other physiological variables such as blood pressure and heart rate.

Other approaches can also be used for anesthesia control. We recently reported successful control of burst suppression using a proportional-integral (PI) controller in simulated rodent [11], simulated human [11], and actual rodent experiments [19]. The experimental studies differed from the ones presented here in that the transitions between target levels were carried out in 5 to 10 minutes ramps. Also, the BSP estimation algorithm in those studies was one rather than two dimensional. Given the stochastic two-dimensional dynamic model and the EEG signal, here we used a stochastic control paradigm consisting of a two-dimensional estimator and an optimal feedback controller in place of the one dimensional estimator and the deterministic PI controller. The model-based two-dimensional state estimator in our framework is one major reason that the current BMI can both make prompt and reliable transitions between levels (median rise time of 49 seconds and fall time of 4.45 minutes) and avoids BSP overshoots and undershoots in any transitions. The stochastic control framework also offers tremendous flexibility. In particular, the MPC allows us to extend the BMI to control with constraints on the control variable and a vital variable such as blood pressure. The stochastic optimal formulation also provides a framework for adjusting the behavior of the BMI by simply modifying the cost function. Finally, while the mathematical derivation for the stochastic framework may be more complex, the final infusion rate solution is straight forward. The LQR solution is given simply by a linear function of the state estimate at each time. The MPC controller optimization problem is convex and can be solved using existing convex optimization software. Indeed we ran the MPC in real-time in our rodent experiments on a standard laptop (Figure 6f).

### A BMI System for Control of Burst Suppression

We chose levels of burst suppression as a control target because it is a physiologically defined brain state [4], [5] with a well-defined EEG signature that can be readily characterized in real time for the purpose of control. The linear two-dimensional state model (Eq. (2)) is the simplest pharmacokinetics representation for relating the concentrations of anesthetic in the blood and in the brain to BSP (Eq. (1)) computed from burst suppression in the EEG. This simplified two-compartment model was sufficient in our experiments to achieve reliable and accurate control of burst suppression.

Our Bayesian state estimator (Eqs. (14)–(17)) computes the central compartment and the effect-site concentrations in real time from the EEG converted into binary observations. Here, both the prior state model (Eq. (6)) and the binomial observation model (Eq. (7)) are non-linear functions of the state. We thus use two approximations at each time step to derive the estimator recursions, a linear approximation to the prior model at that step and a Gaussian approximation to the posterior model. Gaussian Laplace-type approximations have been successfully used in many applications for example in our previous work estimating states with linear prior models from point process observations of neural spiking activity [12], [13], [47]–[50], [56]–[59]. Our system identification procedure used the one-dimensional version of the binary filter, coupled with a non-linear least squares procedure to estimate model parameters (Eq. (2)) for each animal and thereby, implement individually tailored control strategies. Future work can extend this system identification procedure to an efficient expectation-maximization (EM) algorithm by replacing the one-dimensional binary filter algorithm with the current Bayesian state estimator [13], [60], or can design an adaptive estimator that not only computes the BSP but also updates the system parameters during the several hours of real-time control.

We demonstrated in a rodent model that the BMI achieved reliable and accurate control of burst suppression. It would also be valuable as a next step to test this BMI in a rodent model of refractory seizures or intractable intracranial hypertension prior to testing it in humans.

### A BMI System for Control of Medically-Induced Coma and States of General Anesthesia

A BMI system to automatically control medically-induced coma could provide considerable cost-saving and therapeutic benefits. Although the state of medical coma is often required for several days, it is achieved by manually adjusting the anesthetic infusion rate to maintain a specified level of burst suppression assessed by continual visual inspection of the EEG. Automated control would allow much more efficient use of intensive care unit personnel as a single nurse per shift would not have to be solely dedicated to the task of manually managing the drug infusion of a single patient for several days. Hence even assuming the same patient outcomes between automated and manual control, there could be important savings in intensive care unit resources under the automated control regimen.

In addition to the inefficient use of the intensive care unit staff, manual manipulation of the infusion rate does not approximate the infusion rate changes of an automatic controller (Figure 6). Similarly, visual inspection of the EEG does not provide an accurate estimate of the state of burst suppression. The current work establishes the feasibility of implementing automated, accurate and reliable control of medical coma in a rodent model suggesting that a BMI could be developed to study whether such accurate control improves patient outcomes. For example, reliable and accurate control of medical coma could offer the possibility of ensuring adequate brain protection for intracranial hypertension and adequate therapy for status epilepticus while using the least amount of anesthetic and minimizing overshoots when transitioning to a desired level of burst suppression. Reliable and accurate control would also make it easier to induce periodic arousals to conduct neurological assessments and prevent anesthetic overdose syndrome [61]. To establish these potential therapeutic benefits of reliable and accurate control of medical coma, outcome studies in rodent models of intracranial pressure and status epilepticus will be required before proceeding to human investigations.

We have also shown that other states of general anesthesia have well defined EEG signatures [62], [63]. Therefore, the ability of our BMI to track accurately changing target levels of burst suppression further suggests that it could be adapted to control states of general anesthesia and sedation for patients requiring surgical or non-surgical procedures. Our stochastic estimation paradigm and model predictive controller could also be used to control jointly the state of general anesthesia and physiological variables such as blood pressure. These investigations will be the topics of future reports.

## Supporting Information

Figure S1
**Evolution of** 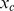 **and** 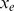 **in real-time BMI experiments.** Each subfigure shows the estimated 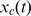 (top panel) and 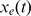 (bottom panel) in the six real-time BMI experiments (Figure 6) using the bounded LQR strategy (a–e) and the MPC strategy (f).(TIF)

Text S1 **Derivation of the update step of the recursive Bayesian estimator and the optimal feedback control solution.**
(PDF)
