# Supplementary figures and images for: A Brain-Machine Interface for Control of Medically-Induced Coma
Source: PLoS Comput Biol. 2013 Oct 31;9(10):e1003284. doi: 10.1371/journal.pcbi.1003284 (PMC3814408; doi:10.1371/journal.pcbi.1003284)

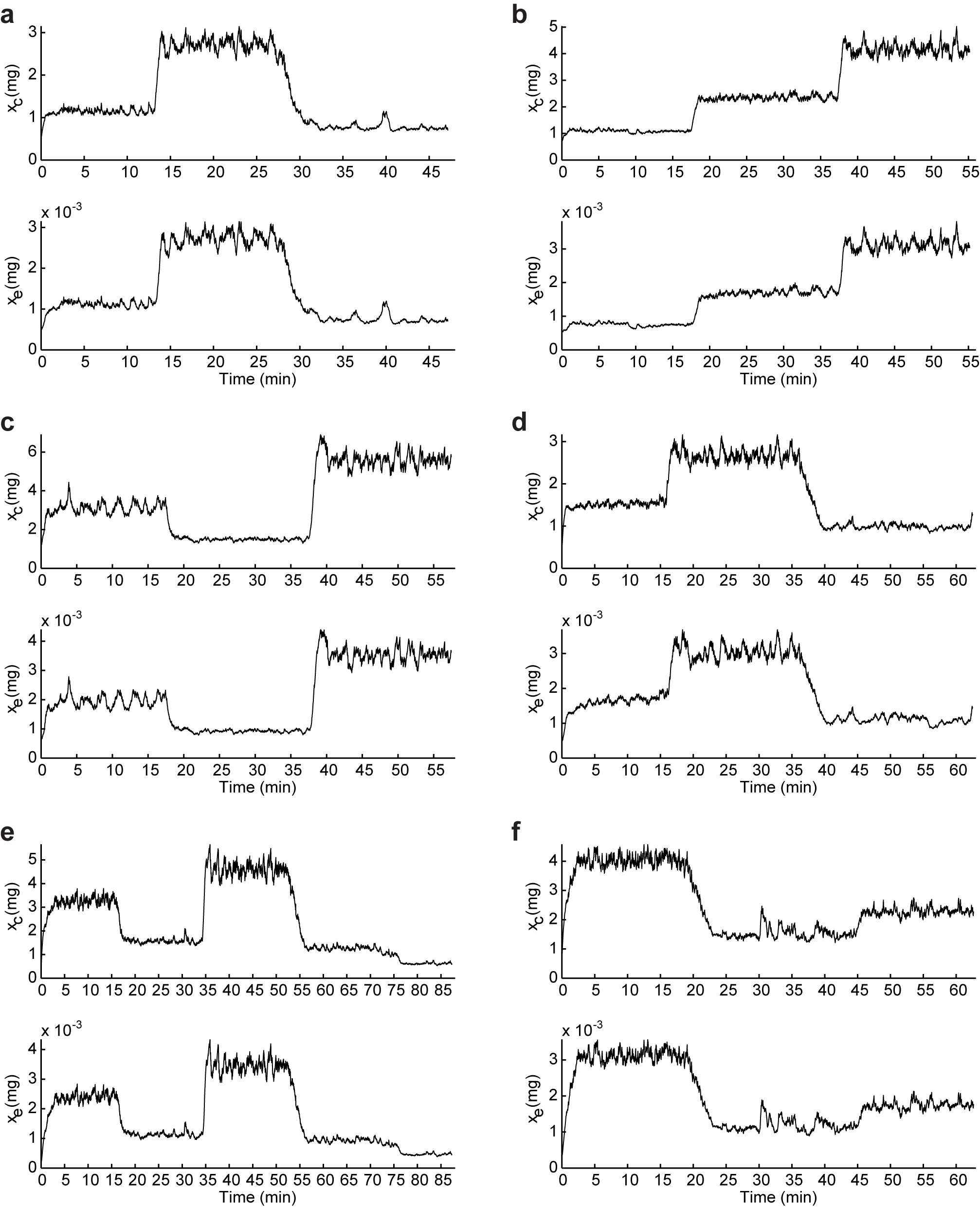

Supplement: Figure S1 — Evolution of and in real-time BMI experiments. Each subfigure shows the estimated (top panel) and (bottom panel) in the six real-time BMI experiments (Figure 6) using the bounded LQR strategy (a–e) and the MPC strategy (f). (TIF) [file pcbi.1003284.s001.tif]
