# Supplementary material for: A Brain-Machine Interface for Control of Medically-Induced Coma
Source: PLoS Comput Biol. 2013 Oct 31;9(10):e1003284. doi: 10.1371/journal.pcbi.1003284 (PMC3814408; doi:10.1371/journal.pcbi.1003284)
Supplement: Text S1 — Derivation of the update step of the recursive Bayesian estimator and the optimal feedback control solution. (PDF) [file pcbi.1003284.s002.pdf]

Text S1
A Brain-Machine Interface for Control of Medically-Induced Coma

Maryam M. Shanechi[4,5*†], Jessica J. Chemali[1,†], Max Liberman[1], Ken Solt[1], Emery N. Brown[1−3*]
1 Department of Anesthesia, Critical Care and Pain Medicine, Massachusetts General Hospital, Boston, MA, USA
2 Institute for Medical Engineering and Science, Massachusetts Institute of Technology, Cambridge, MA, USA
3 Department of Brain and Cognitive Sciences, Massachusetts Institute of Technology, Cambridge, MA, USA
4 School of Electrical and Computer Engineering, Cornell University, Ithaca, NY, USA
5 Department of Electrical Engineering and Computer Science, University of California, Berkeley, CA, USA
† These authors contributed equally to this work.
∗ E-mail: shanechi@cornell.edu, enb@neurostat.mit.edu

Update Step Derivation

To derive the update step of the estimator, we make a Gaussian approximation to the posterior density at each time step. As explained in the main text, given this Gaussian approximation the prediction density at a given time step will also be approximately Gaussian. Using these Gaussian approximations and taking the logarithm of (8) in the main text we get

$$\log(p(\mathbf{z}_t|N_{1:t})) = R + N_t \log(p_t(\mathbf{z}_t)) + (N - N_t)\log(1 - p_t(\mathbf{z}_t)) + \log(p(\mathbf{z}_t|N_{1:t-1})) \tag{S.1}$$

$$\Rightarrow -\frac{1}{2}(\mathbf{z}_t - \mathbf{z}_{t|t})^T \mathbf{W}_{t|t}^{-1}(\mathbf{z}_t - \mathbf{z}_{t|t}) = N_t \log(p_t(\mathbf{z}_t)) + (N - N_t)\log(1 - p_t(\mathbf{z}_t)) \tag{S.2}$$

$$-\frac{1}{2}(\mathbf{z}_t - \mathbf{z}_{t|t-1})^T \mathbf{W}_{t|t-1}^{-1}(\mathbf{z}_t - \mathbf{z}_{t|t-1}) + C \tag{S.3}$$

where R and C are constants (i.e., not a function of $\mathbf{z}_t$). Differentiating the above expression with respect to $\mathbf{z}_t$ we get

$$-\mathbf{W}_{t|t}^{-1}(\mathbf{z}_t - \mathbf{z}_{t|t}) = N_t \frac{\partial p_t(\mathbf{z}_t)}{\partial \mathbf{z}_t}\frac{1}{p_t(\mathbf{z}_t)} - (N - N_t)\frac{\partial p_t(\mathbf{z}_t)}{\partial \mathbf{z}_t}\frac{1}{1 - p_t(\mathbf{z}_t)} - \mathbf{W}_{t|t-1}^{-1}(\mathbf{z}_t - \mathbf{z}_{t|t-1}) \tag{S.4}$$

$$= \frac{\partial p_t}{\partial \mathbf{z}_t}\frac{1}{p_t(1 - p_t)}(N_t - N p_t) - \mathbf{W}_{t|t-1}^{-1}(\mathbf{z}_t - \mathbf{z}_{t|t-1}) \tag{S.5}$$

Evaluating at $\mathbf{z}_{t|t-1}$ we get the update step

$$\mathbf{z}_{t|t} = \mathbf{z}_{t|t-1} + \mathbf{W}_{t|t}\begin{bmatrix} 0 \\ \frac{\partial p_t}{\partial z_e(t)}\frac{1}{p_t(1-p_t)}(N_t - N p_t) \end{bmatrix}_{\mathbf{z}_{t|t-1}} \tag{S.6}$$

where $[\cdot]_{\mathbf{z}_{t|t-1}}$ indicates the evaluation of the inside expression at $\mathbf{z}_{t|t-1}$ and

$$\frac{\partial p_t}{\partial z_e(t)} = \frac{\partial p_t}{\partial x_e(t)}\frac{\partial x_e(t)}{\partial z_e(t)} \tag{S.7}$$

$$= \frac{x_e(t)\exp(x_e(t))}{1 + \exp(x_e(t))}(1 - p_t) \tag{S.8}$$

$$= c_t. \tag{S.9}$$

Note that we have used the identity $z_e(t) = \log x_e(t)$. Now differentiating again we get the posterior covariance

$$\mathbf{W}_{t|t}^{-1} = \mathbf{W}_{t|t-1}^{-1} + \begin{bmatrix} 0 & 0 \\ 0 & \gamma_t \end{bmatrix}_{\mathbf{z}_{t|t-1}} \tag{S.10}$$

where

$$\gamma_t = -\frac{\partial}{\partial z_e(t)} \left(\frac{\partial p_t}{\partial z_e(t)} \frac{1}{p_t(1-p_t)} (N_t - N p_t) \right) \tag{S.11}$$

$$= \frac{N c_t^2}{p_t(1-p_t)} - \frac{N_t - N p_t}{p_t(1-p_t)} \left[\frac{\partial^2 p_t}{\partial z_e^2(t)} - \frac{1-2p_t}{p_t(1-p_t)} c_t^2 \right] \tag{S.12}$$

where

$$\frac{\partial^2 p_t}{\partial z_e^2(t)} = c_t \left[1 + x_e(t) - (1-p_t) x_e(t) \exp(x_e(t)) \right] \tag{S.13}$$

Optimal Feedback-Controller Derivation

The controller's goal is to derive the state close to a non-zero target concentration value. Namely, in the control of burst suppression the goal is to achieve a desired non-zero target BSP level, p^*, or equivalently a non-zero effect-site concentration $x^* = \log\left((1+p^*)/(1-p^*)\right)$. To find the solution, we thus shift the origin of the state-space to x^* [14] . This way, the control goal is equivalent to deriving the shifted state variable close to zero, as in the classical LQR formulation.

To shift the origin, if we find a valid solution for u^* for which

$$\mathbf{x}^* = \mathbf{A}\mathbf{x}^* + \mathbf{B}u^*, \qquad \mathbf{x}^* = \begin{bmatrix} g(x^*) \\ x^* \end{bmatrix} \tag{S.14}$$

where g is a function to be solved for, then making a change of variables, $\tilde{\mathbf{x}}_t = \mathbf{x}_t - \mathbf{x}^*$ and $\tilde{u}_t = u_t - u^*$ we can write using (2) in the main text

$$\tilde{\mathbf{x}}_{t+1} + \mathbf{x}^* = \mathbf{A}(\tilde{\mathbf{x}}_t + \mathbf{x}^*) + \mathbf{B}(\tilde{u}_t + u^*), \tag{S.15}$$

which using (S.14) is simplified to

$$\tilde{\mathbf{x}}_{t+1} = \mathbf{A}\tilde{\mathbf{x}}_t + \mathbf{B}\tilde{u}_t. \tag{S.16}$$

Hence the shifted variables satisfy the state model in (2) in the main text. Note that u^* is the constant control input that will keep the system in (2) in the main text at the effect-site concentration value x^*. Since we have one degree of freedom u^* and need to set one variable $x_e = x^*$, there exists a solution to (S.14) given by

$$g(x^*) = \frac{k_{ec}}{k_{ce}} x^*, \qquad u^* = \frac{k_{c0} k_{ec}}{k_{ce}} x^* \tag{S.17}$$

Now in terms of the shifted variables we are back to the classical LQR form since we can write the controller cost in our problem in terms of the shifted state variables as

$$J = \sum_{t=1}^{T-1} \left(\tilde{\mathbf{x}}_t' \mathbf{Q} \tilde{\mathbf{x}}_t + w_r \tilde{u}_t^2 \right) + \tilde{\mathbf{x}}_T' \mathbf{Q}_T \tilde{\mathbf{x}}_T, \tag{S.18}$$

with $\mathbf{Q}$ and w_r selected appropriately for a desired controlled response (as will be discussed in the Results section). We can now solve for the optimal $\tilde{u}_t$ using (22) in the main text, and the optimal infusion rate at each time is then given by $\tilde{u}_t + u^*$. Using the steady-state LQR solution this optimal drug infusion rate is given by

$$u_t = -\mathbf{L}(\mathbf{x}_t - \mathbf{x}^*) + u^* \tag{S.19}$$

Hence (23) in the main text, (S.17), and (S.19) provide the controller's drug infusion rate at time t given the current state $\mathbf{x}_t$, which is provided by the estimator.
